# Angle-Insensitive Broadband Absorption Enhancement of Graphene Using a Multi-Grooved Metasurface

**DOI:** 10.1186/s11671-019-2937-7

**Published:** 2019-03-20

**Authors:** Tian Sang, Jian Gao, Xin Yin, Honglong Qi, La Wang, Hongfei Jiao

**Affiliations:** 1 Department of Photoelectric Information Science and Engineering, School of Science, Jiangnan University, Wuxi, 214122 China; 2 Key Laboratory of Advanced Micro-Structured Materials MOE, Institute of Precision Optical Engineering, School of Physics Science and Engineering, Tongji University, Shanghai, 200092 China

**Keywords:** Graphene, Light absorption, Broadband, Angle-insensitive, Multi-grooved metasurface

## Abstract

An angle-insensitive broadband absorber of graphene covering the whole visible spectrum is numerically demonstrated, which is resulted from multiple couplings of the electric and magnetic dipole resonances in the narrow metallic grooves. This is achieved by integrating the graphene sheet with a multi-grooved metasurface separated by a polymethyl methacrylate (PMMA) spacer, and an average absorption efficiency of 71.1% can be realized in the spectral range from 450 to 800 nm. The location of the absorption peak of graphene can be tuned by the groove depth, and the bandwidth of absorption can be flexibly controlled by tailoring both the number and the depth of the groove. In addition, broadband light absorption enhancement of graphene is robust to the variations of the structure parameters, and good absorption properties can be maintained even the incident angle is increased to 60°.

**Electronic supplementary material:**

The online version of this article (10.1186/s11671-019-2937-7) contains supplementary material, which is available to authorized users.

## Background

Graphene has been demonstrated as a good candidate for optoelectronic devices because of its remarkable electronic, mechanical and tunable optical properties [1–3]. For many applications such as photo detections and solar cells, strong absorption of graphene is desired in order to generate a large amount of electron-hole pairs and produce a large photocurrent [4, 5]. From the terahertz to mid-infrared ranges, graphene behaves like a metal and can be functioned as good absorber due to its strong plasmonic response [6–8]. On the contrary, in the visible and near-infrared regions, graphene exhibits a nearly wavelength-independent absorption of about 2.3% at normal incidence [9], which seriously limits its further application in photoelectric detection.

In recent years, various approaches have been suggested to enhance light absorption of graphene in the visible and near-infrared regions, and the physical mechanisms behind the absorption enhancement of graphene include epsilon-near-zero effect [10], cavity resonance [11–13], attenuated total reflectance [14], guided-mode resonance [15–18], critical coupling [19–21], Fano resonance [22, 23], plasmonic resonance [24–26], and magnetic resonance [27–29]. Unfortunately, the bandwidths of those absorbers are generally narrow due to their resonance nature. Very recently, it is shown that the absorption bandwidth of graphene can be extended by increasing the light absorption channels [30–35]. On the one hand, by using the patch resonator [30] or the Ag nanodisk arrays [31], dual-band light absorption enhancement of graphene can be achieved. More light absorption channels of graphene can be realized by increasing the thickness of the waveguide [32], and broadband absorption enhancement of graphene is possible by using multiple Ag nanodisk arrays [33]. On the other hand, the angular absorption channels of graphene can be increased by using attenuated-total-reflection configuration [34], and angularly dense comb-like enhanced absorption of graphene can be obtained by the excitation of guided-mode resonance of one-dimensional photonic crystals [35]. In real applications, the enhancement of light-graphene coupling in a wide spectral range is very important for devices such as photodetectors and photovoltaics. However, there are only few researches on broadband absorption enhancement of graphene in the visible and near-infrared regions, and angle-insensitive broadband absorbers of graphene covering the whole visible region are highly desired.

In this work, a novel angle-insensitive broadband absorber of graphene covering the whole visible region is proposed by integrating the graphene sheet with a multi-grooved metasurface. The enhanced absorption band of graphene has arisen from the multiple couplings of electric and magnetic dipole resonances confined in the groove cavity. The absorption band of graphene can be flexibly controlled by tailoring both the number and the depth of the grooves. High absorption efficiency can be maintained even if the structure parameters and the incident angle are significantly altered.

## Methods

**Fig. 1:**
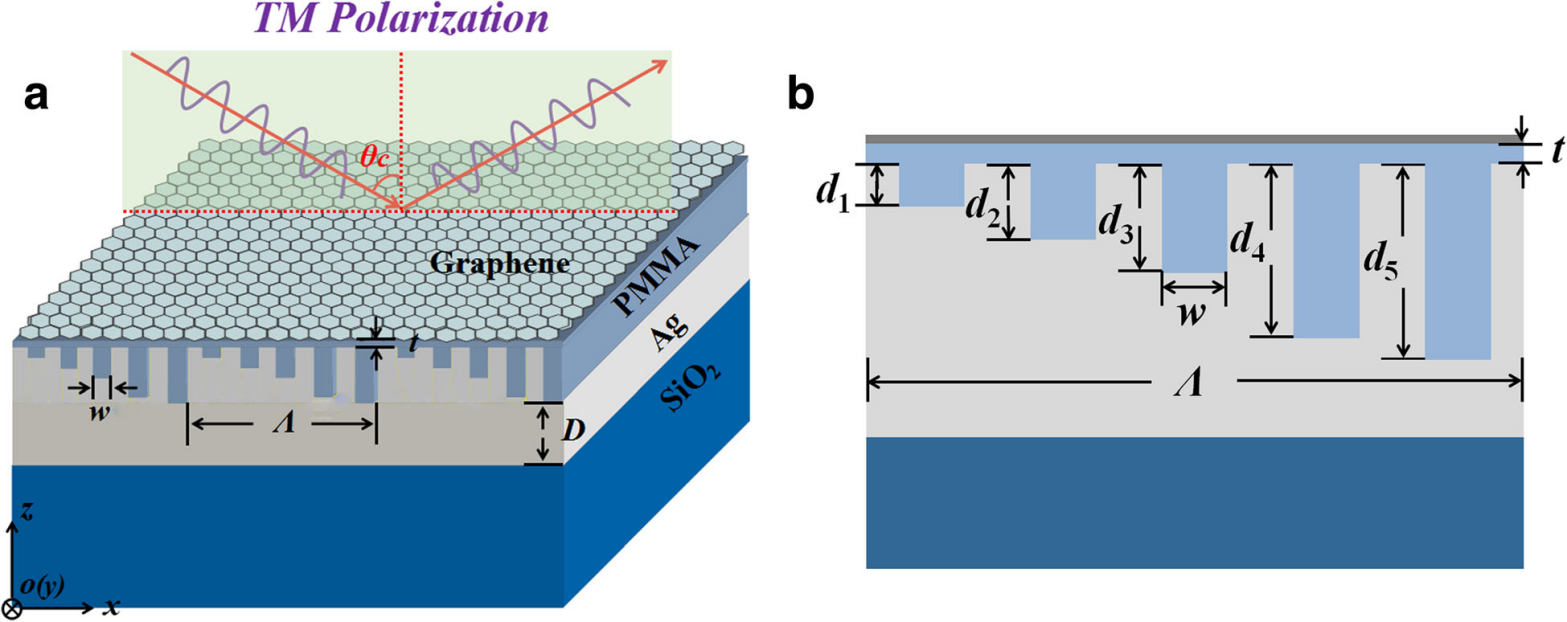
**a** Schematic diagram of the multi-grooved metasurface for angle-insensitive broadband absorption of graphene. **b** Cross-section diagram of a unit cell of the structure

Figure 1 shows a schematic diagram of the multi-grooved metasurface illuminated by the TM plane wave (magnetic-field vector lies along the *y*-axis) for angle-insensitive broadband absorption enhancement of graphene. The unit cell of the structure consists of a planar graphene sheet and a patterned silver film with five grooves separated by a polymethyl methacrylate (PMMA) spacer. The PMMA layer is functioned as a buffer layer which controls the coupling between graphene and the patterned silver film, and it can also be easily transferred onto the multigrooved surface by spin-coating in application. The period of the unit cell is *Λ*, the thickness of the PMMA spacer is *t*, the thickness of the bottom silver film is *D*, and the substrate is silica. The geometry of the groove is described by both its width *w* and its depth. The width of the five grooves is equal, and their depths are *d*_1_, *d*_2_, *d*_3_, *d*_4_, and *d*_5_, respectively. The refractive index of PMMA is 1.49 [36], and the complex refractive indices of the silver film are taken from Palik [37]. The planar graphene sheet consists of N layers of monolayer graphene, and the thickness of the graphene sheet is 3.4 nm as *N* = 10 [11, 27]. The monolayer graphene is modeled as an infinitesimally thin surface with the surface conductivity *σ*_*g*_ calculated from Kubo formula [38, 39]. At finite temperature, it can be divided into intra- and interband contributions:

1 $$ {\sigma}_g\left(\omega \right)={\sigma}_{\mathrm{intra}}\left(\omega \right)+{\sigma}_{\mathrm{inter}}\left(\omega \right) $$

2 $$ {\sigma}_{\mathrm{intra}}\left(\omega \right)=-j\frac{e^2{k}_BT}{\pi {\mathrm{\hslash}}^2\left(\omega -2j\Gamma \right)}\left[\frac{\mu_c}{k_BT}+2\mathrm{l}n\left({e}^{-\frac{\mu_c}{k_BT}}+1\right)\right] $$

3 $$ {\sigma}_{\mathrm{inter}}\left(\omega \right)=-j\frac{e^2}{4\pi \mathrm{\hslash}}\mathrm{l}n\left[\frac{2\left|{\mu}_c\right|-\left(\omega -j2\Gamma \right)\mathrm{\hslash}}{2\left|{\mu}_c\right|+\left(\omega -j2\Gamma \right)\mathrm{\hslash}}\right] $$

where *e* and *ħ* are the elementary charge and reduced Planck’s constant, respectively. *k*_*B*_ is the Boltzmann constant, *μ*_*c*_ is the chemical potential, *Γ* = 1/2*τ* is the phenomenological scattering rate, and *τ* is the momentum relaxation time. The physical parameters of the graphene are set as *μ*_*c*_ = 0.15 eV, *T* = 300 K, and *τ* = 0.50 ps.

In simulations, the finite-difference time-domain (FDTD) method (Lumerical FDTD solutions) is adopted to calculate the absorption properties of the graphene-based multi-grooved metasurface. Periodic boundary conditions (PBCs) are employed in the *x* directions, while boundaries in the *z* direction are adopted as perfectly matched layers (PMLs). Reflectivity (*R*) and transmissivity (*T*) are obtained by two monitors at the top and bottom of the structure. The bottom silver film is chosen to be optically thick enough (*D* = 100 nm) to prevent light transmission; therefore, total absorption (*A*) of the structure can be reduced as *A* = 1–*R*. The absorption of graphene (*A*_*g*_) can be calculated as [24]:

4 $$ {A}_g=\left[{P}_{\mathrm{up}}\left(\lambda \right)-{P}_{\mathrm{down}}\left(\lambda \right)\right]/{P}_{\mathrm{in}}\left(\lambda \right) $$

where *P*_up_ (*λ*) and *P*_down_ (*λ*) are the powers passing through the upside and downside planes of the graphene sheet at the wavelength *λ*, respectively. *P*_in_ (*λ*) represents the incident power at the wavelength *λ*. In simulation, *P*_in_ (*λ*) is the power of the light source, and two power monitors are inserted at the top and bottom planes of the graphene to obtain *P*_up_ (*λ*) and *P*_down_ (*λ*). These powers are extracted from the total field in the FDTD simulations.

## Results and Discussions

**Fig. 2:**
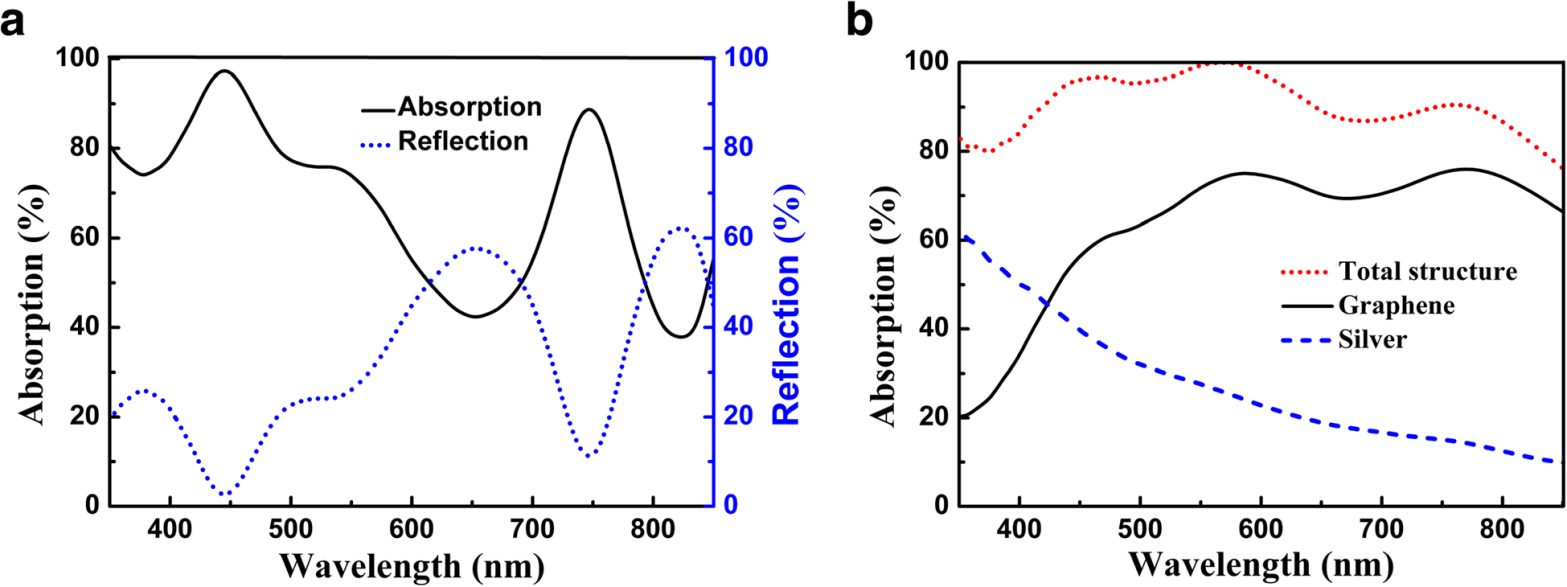
**a** Spectra of the multi-grooved metasurface without graphene. **b** Absorption spectra of the total structure, graphene, and silver for the multi-grooved metasurface with graphene. The parameters are *Λ* = 300 nm, *t* = 5 nm, *w* = 30 nm, *D* = 100 nm, *d*_1_ = 20 nm, *d*_2_ = 35 nm, *d*_3_ = 50 nm, *d*_4_ = 80 nm, *d*_5_ = 90 nm, *N* = 10, and *θ*_*c*_ = 0°

Figure 2 shows spectral response of the multi-grooved metasurface without and with graphene. The structure parameters, such as the number of groove, the depth and width of groove, and the thickness of the PMMA spacer, are optimized so as to obtain broadband absorption enhancement in the visible region. As can be seen in Fig. 2a, the multi-grooved metasurface without graphene can be functioned as a plasmonic absorber, and light absorption can be enhanced in the visible region due to the surface plasmon effect of the nanostructured silver film. See Fig. 2b for the multi-grooved metasurface with graphene, and light absorption can be significantly enhanced in the whole visible region. The average absorption of the total structure reaches 92.7% over the wavelength range of 400–800 nm, which is comparable with many plasmonic absorbers, both in absorption efficiency and absorption bandwidth [40–43]. Interestingly, the light energy is mainly dissipated in graphene rather than in silver. The absorption efficiency of graphene is significantly enhanced in an extended wavelength region, and its average absorption efficiency reaches 71.1% in the spectral range from 450 to 800 nm. However, because the surface plasmon mode can only be excited by the TM polarization, there is no obvious absorption enhancement for the multi-grooved metasurface under the TE wave illumination (see Additional file 1: Figure S1).

**Fig. 3:**
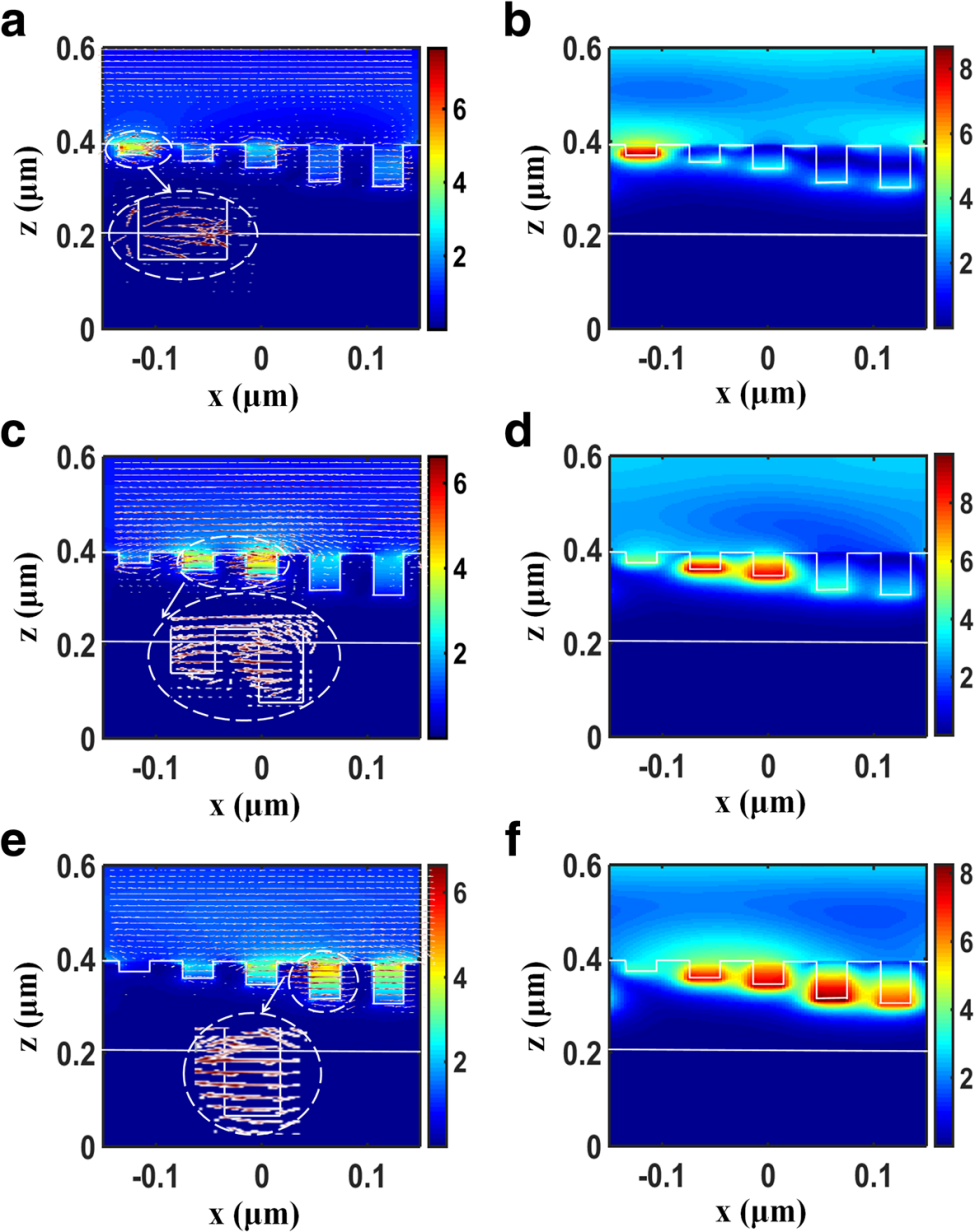
Normalized distributions of electric and magnetic fields of the unit cell of the structure at the wavelengths of 450 nm for (**a**) and (**b**); 600 nm for (**c**) and (**d**); 750 nm for (**e**) and (**f**). The inserted white dash area is the enlarged view of the grooves, and red arrows indicate the direction of the electric field. The structure parameters are the same as in Fig. 2

To gain insights into the effect of broadband absorption enhancement of graphene under the TM wave illumination, the electric and magnetic field distributions of the structure for different wavelengths are investigated. As can been seen in Fig. 3, the electric field is highly concentrated and enhanced around the corner of the metallic groove, and its direction is nearly parallel to the *x*-axis, corresponding to an electric dipole resonance mode [44, 45]. On the contrary, the magnetic field is strongly enhanced in the cavity of the metallic groove, and its direction is perpendicular to the *xoz*-plane, corresponding to a magnetic dipole resonance mode [26, 46]. The electromagnetic coupling of the electric and magnetic dipole resonances in the metallic grooves remarkably increases the light-graphene interaction, resulting in enhanced light absorption of graphene. Note the location of field enhancement is mainly concentrated in the shallower groove for short wavelength, and it shifts to deeper groove as wavelength is increased; thus multiple couplings of the electric and magnetic dipole resonances can be supported for the multi-grooved structure with different groove depths, resulting in broadband light absorption of graphene which covers the whole visible region.

**Fig. 4:**
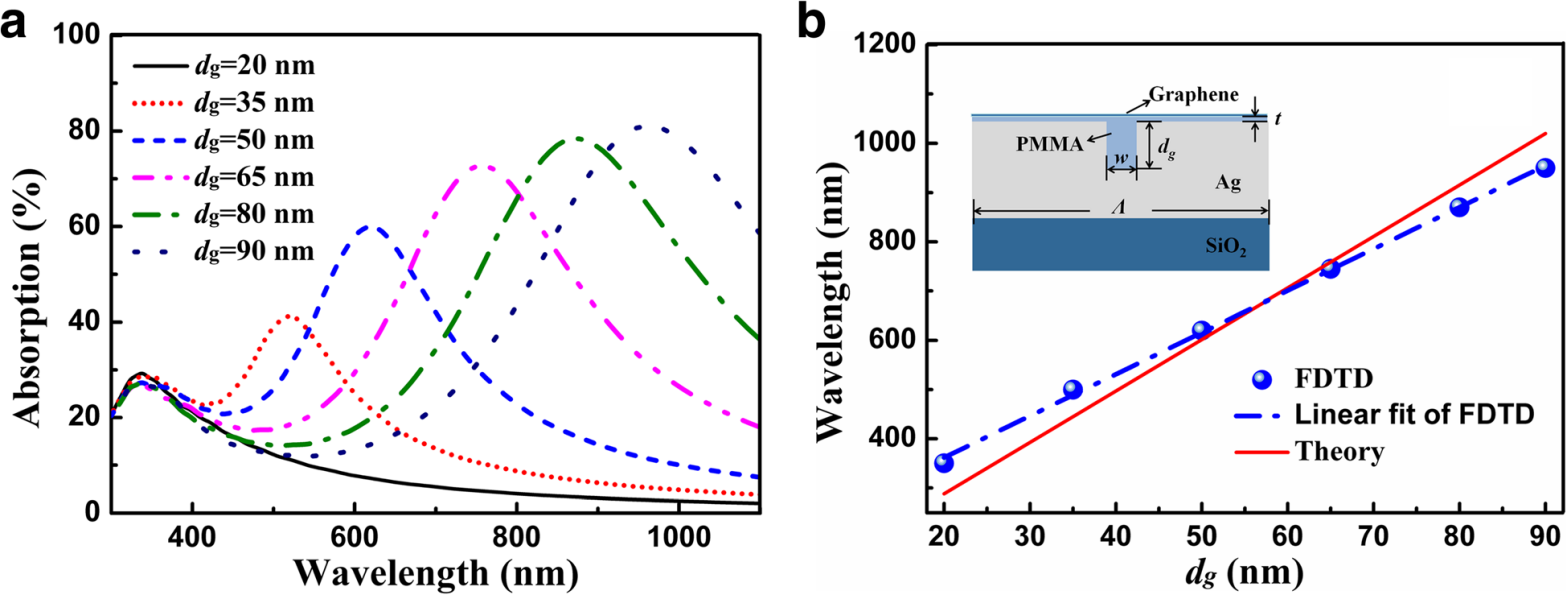
Absorption response of graphene for the single-grooved structure as shown in the figure inset. **a** Absorption response of graphene as a function of the groove depth. **b** FDTD result of the location of absorption peak of graphene as a function of groove depth, and theoretical result of resonance wavelength as a function of groove depth. The parameters are *Λ* = 300 nm, *t* = 5 nm, *N* = 10, and *w* = 30 nm

To further identify the location of the absorption peak of graphene of the multi-grooved metasurface, resonant properties of the single-grooved structure is studied. For the single-grooved structure shown in the inset of Fig. 4b, the resonance wavelength of the groove cavity under TM polarization is given as [47]:

5 $$ 2{n}_{\mathrm{eff}}{d}_g+\frac{1}{2}\lambda = M\lambda, $$

where *M* is the mode number, and *M* = 1 in calculation; *n*_eff_ is the effective refractive index of the groove cavity, which can be equivalent to the mode refractive index of the metal-insulator-metal (MIM) waveguide. Only the fundamental mode of TM_0_ can be supported because the groove width is far smaller than wavelength, and the corresponding *n*_eff_ can be determined by using the even mode dispersion of the MIM waveguide [48]:

6 $$ \tanh \left(\frac{w\sqrt{\beta^2-{k}_0^2{\varepsilon}_d}}{2}\right)=-\frac{\varepsilon_d\sqrt{\beta^2-{k}_0^2{\varepsilon}_m}}{\varepsilon_m\sqrt{\beta^2-{k}_0^2{\varepsilon}_d}}, $$

where *ε*_*d*_ and *ε*_*m*_ are the dielectric constants of PMMA and silver, respectively; *k*_0_ is the wave vector of incident light, *β* is the propagation constant of the MIM waveguide mode, and *n*_eff_ = *β*/*k*_0_.

As can be seen in Fig. 4a, for the single-grooved structure, the absorption efficiency of graphene is increased as the groove depth is increased, and the absorption peak of graphene is shifted to the longer wavelength as well. As can be seen in Fig. 4b, the locations of absorption peaks of graphene are in good agreement with the theoretical results of the resonance wavelength of the groove cavity. The slope of the FDTD result is 8.48, which is close to the slope of the theoretical result of 10.46. According to Eq. (5), the location of absorption peak of graphene is redshifted with the increase of the groove depth, and it covers the whole visible region as the groove depth is varied within the range of 20–90 nm. Therefore, the location of the absorption peak of graphene can be tuned by the groove depth, and broadband absorption of graphene can be realized if multiple grooves with different groove depths are integrated into the unit cell of the structure, which further verifies the physical mechanism of broadband light absorption of graphene for the multi-grooved metasurface. However, for a fixed period and a fixed groove width, it does not mean that the more the number of the groove is, the better the absorption performance of graphene will be (see Additional file 1: Figure S2). Thus, the absorption performance of graphene can be flexibly controlled by tailoring both the number and the depth of the groove for the multi-grooved configuration.

**Fig. 5:**
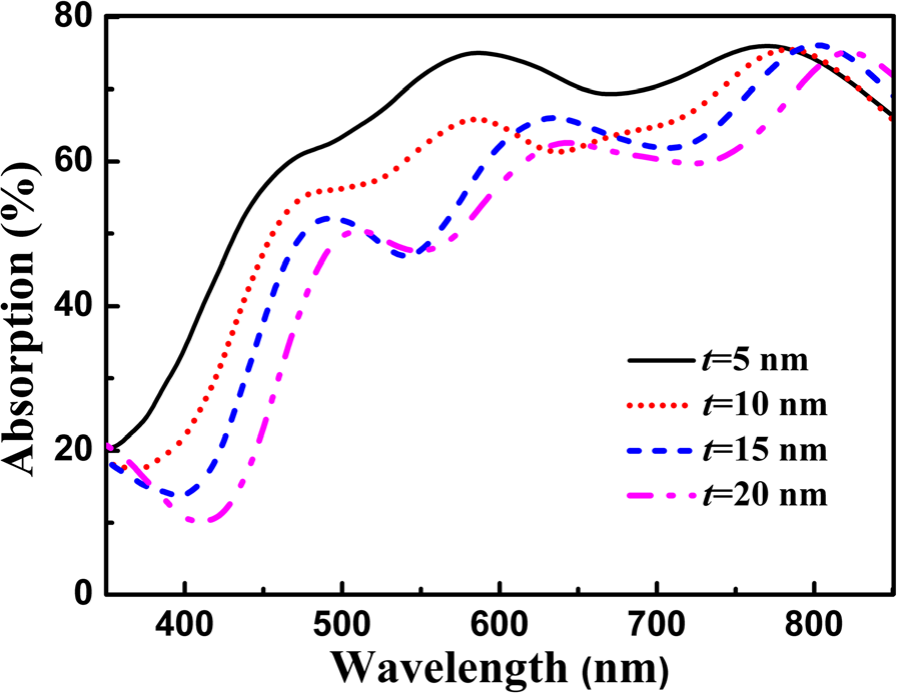
Absorption response of graphene as a function of the thickness of the spacer layer for the multi-grooved structure, and other parameters are the same as in Fig. 2

To further evaluate the absorption performance of graphene integrated with the multi-grooved metasurface, we first investigated the influence of the thickness of the spacer layer on light absorption of graphene. As can be seen in Fig. 5, the absorption response of graphene is robust to the variation of the thickness of the spacer layer, and the broad absorption band can be maintained as the thickness of the spacer layer is increased from 5 nm to 20 nm. As the thickness of the spacer layer is increased, the absorption band of the graphene shifts to the longer wavelength due to the increase of the optical thickness of the structure. In addition, because the spacer layer possesses the function of the buffer layer, which controls the electromagnetic coupling between the metallic groove and graphene, the average absorption efficiency of the graphene is decreased with the increase of the thickness of the spacer layer.

**Fig. 6:**
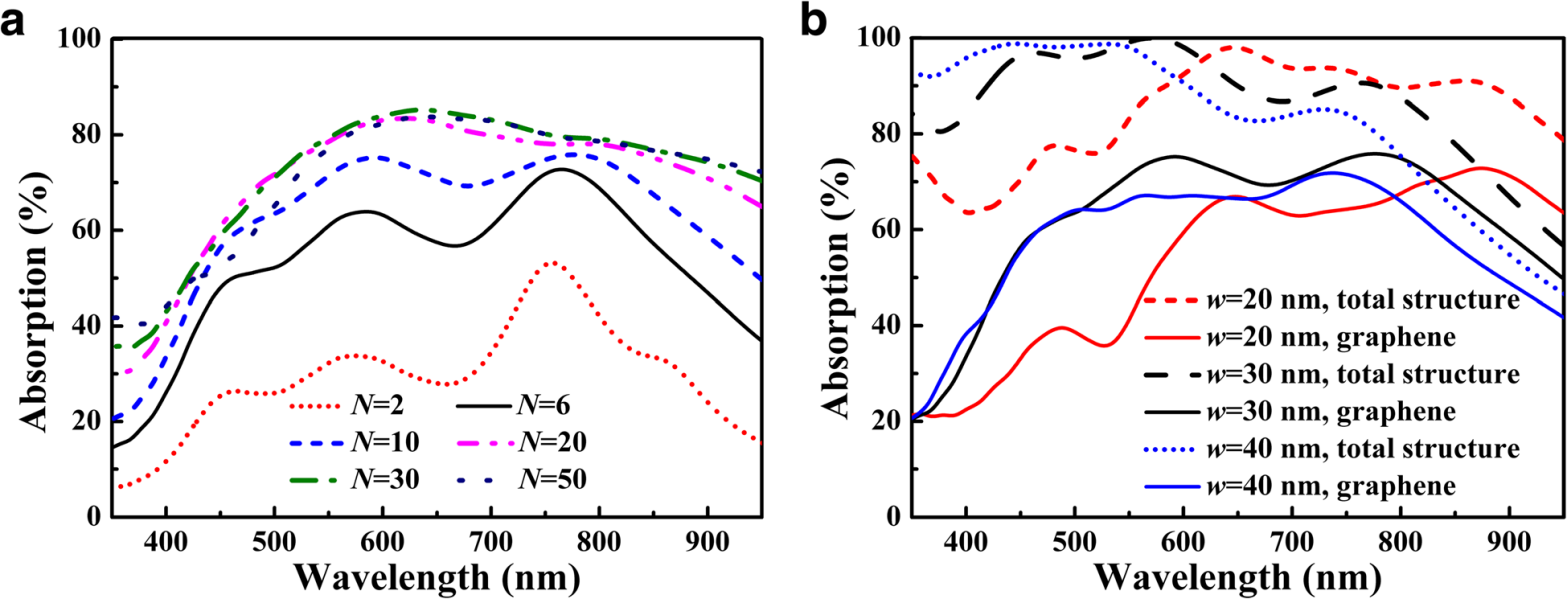
**a** Absorption response of graphene as a function of the number of monolayer graphene. **b** Absorption spectra of the total structure and graphene as functions of the groove width with *N* = 10. Other parameters are the same as in Fig. 2

Figure 6 shows the influence of the number of monolayer graphene and the groove width on light absorption of graphene, and it can be seen that the absorption performance of graphene is robust to the variations of both *N* and *w*. In Fig. 6a, light absorption of graphene can be remarkably enhanced as the number of monolayer graphene is increased to 10; however, the overall absorption enhancement slows down for *N* > 10 and it becomes saturated as *N* is increased to 30. Light absorption of graphene is not always increased with the increase of the number of monolayer graphene, and similar phenomenon can also be observed in the graphene-based waveguide-resonance gratings [49]. In Fig. 6b, it can be seen that the absorption band is blueshifted as the groove width is increased, and the average absorption attains its maximum at the design value of *w* = 30 nm for both the total structure and graphene in the visible region. Because the electromagnetic coupling of the electric and magnetic dipole resonances is mainly confined in the groove, deviation from the design value of groove width with ± 10 nm will distinctly affect the absorption performance of the multi-grooved metasurface.

**Fig. 7:**
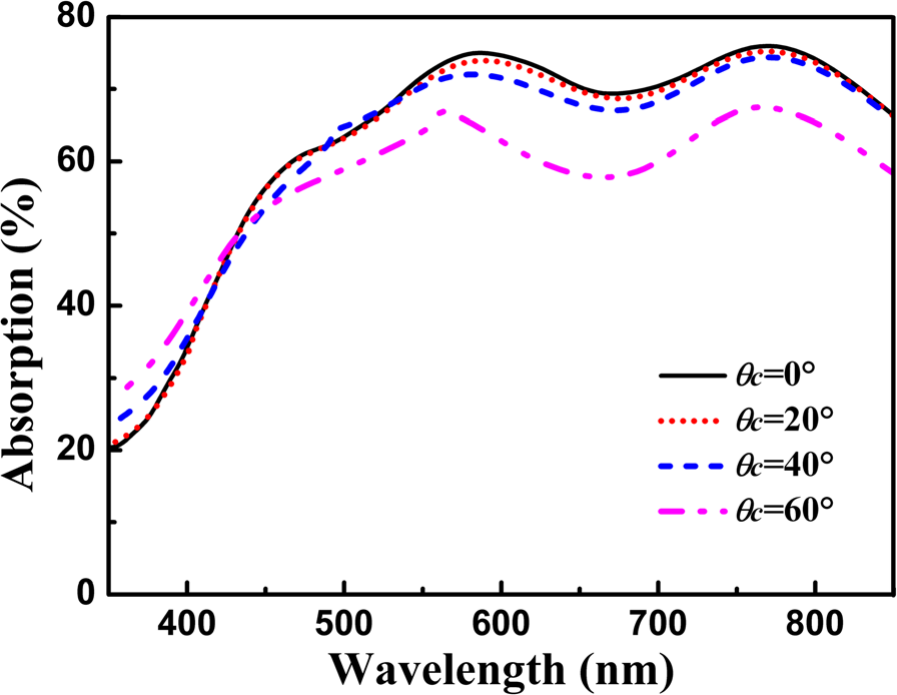
Absorption response of graphene as a function of the incidence angle for the multi-grooved structure, and other parameters are the same as in Fig. 2

We also investigate the angular robustness of the proposed graphene absorber integrated with the multi-grooved metasurface. In Fig. 7, one can find that the absorption response of graphene is robust to the variation of the incident angle. It can be calculated that an average absorption efficiency of 61.5% can be achieved even at *θ*_*c*_ = 60° within the spectral range of 450–800 nm, and the absorption band is kept almost the same although the incident angle is significantly altered. This is because that the broadband absorption enhancement of graphene integrated with the multi-grooved metasurface is originated from the coupling of the electric and magnetic dipole resonances in the groove cavity, which is almost immune to the variation of the incident angle. The angle-insensitive absorption performances are very important because the absorption performances of most graphene-based absorbers are generally depended on the incident angle [12–25, 28–35]. Differing from the previous graphene-based absorbers, the proposed structure possesses broad absorption band and angle-insensitive performance simultaneously, which is highly desired in a variety of areas such as omnidirectional absorbers.

## Conclusions

In conclusion, an angle-insensitive broadband absorber of graphene integrated with a multi-grooved metasurface is proposed and its light absorption property is numerically investigated. The absorption band of graphene covers the whole visible region, and an average absorption efficiency of 71.1% can be realized in the spectral range from 450 to 800 nm. The extended absorption band of graphene has arisen from the multiple couplings of electric and magnetic dipole resonances confined in the groove cavity, and its mechanism can be verified by using the single-grooved structure. The location of the absorption peak of graphene can be tuned by the groove depth, and the absorption bandwidth of graphene can be flexibly controlled by tailoring both the number and the depth of the groove. Broadband absorption properties of graphene are almost unaffected by the variation of the thickness of the spacer layer, the number of monolayer graphene, and the groove width. In particular, the light absorption spectra of graphene remain almost the same even at large angles. The idea of using multi-grooved metasurface to widen the interaction band between light and graphene could be also adopted in near-infrared region and other graphene-based optoelectronic devices.

## Additional file

Additional file 1: **Figure S1.** Absorption spectra of the total structure, graphene, and silver for the multi-grooved metasurface with graphene under the TE wave illumination. Figure S2 Absorption spectra for the graphene-based metasurface with different number of groove. (DOCX 380 kb)

## Abbreviations

FDTD: Finite-difference time-domain
MIM: Metal-insulator-metal
PBCs: Periodic boundary conditions
PMLs: Perfectly matched layers
PMMA: Polymethyl methacrylate

## References

[1] Bonaccorso F, Sun Z, Hasan T, Ferrari AC (2010). Graphene photonics and optoelectronics. Nat Photon.

[2] He X, Gao P, Shi W (2016). A further comparison of graphene and thin metal layers for plasmonics. Nanoscale.

[3] He X, Liu F, Lin F, Shi W (2018). Graphene patterns supported terahertz tunable plasmon induced transparency. Opt Express.

[4] Koppens FHL, Mueller T, Avouris P, Ferrari AC, Vitiello MS, Polini M (2014). Photodetectors based on graphene, other two-dimensional materials and hybrid systems. Nat Nanotechnol.

[5] Echtermeyer TJ, Britnell L, Jasnos PK, Lombardo A, Gorbachev RV, Grigorenko AN, Geim AK, Ferrari AC, Novoselov KS (2011). Strong plasmonic enhancement of photovoltage in graphene. Nat Commun.

[6] Xu Z, Wu D, Liu Y, Liu C, Yu Z, Yu L, Ye H (2018). Design of a tunable ultra-broadband terahertz absorber based on multiple layers of graphene ribbons. Nanoscale Res Lett.

[7] de AFJG (2014). Graphene plasmonics: challenges and opportunities. ACS Photon.

[8] He X, Xiao G, Liu F, Lin F, Shi W (2019). Flexible properties of THz graphene bowtie metamaterials structures. Opt Mater Express.

[9] Nair RR, Blake P, Grigorenko AN, Novoselov KS, Booth TJ, Stauber T, Peres NMR, Geim AK (2008). Fine structure constant defines visual transparency of graphene. Science.

[10] Lee S, Tran TQ, Kim M, Heo H, Heo J, Kim S (2015). Angle- and position-insensitive electrically tunable absorption in graphene by epsilon-near-zero effect. Opt Express.

[11] Zhang HJ, Zheng GG, Chen YY, Xu LH (2018). Broadband and wide angle near-unity absorption in graphene-insulator-metal thin film stacks. Superlattice Microst.

[12] Liu JT, Liu NH, Li J, Li XJ, Huang JH (2012). Enhanced absorption of graphene with one-dimensional photonic crystal. Appl Phys Lett.

[13] Furchi M, Urich A, Pospischil A, Lilley G, Unterrainer K, Detz H, Klang P, Andrews AM, Schrenk W, Strasser G, Mueller T (2012). Microcavity-integrated graphene photodetector. Nano Lett.

[14] Pirruccio G, Moreno LM, Lozano G, Rivas JG (2013). Coherent and broadband enhanced optical absorption in graphene. ACS Nano.

[15] Grande M, Vincenti MA, Stomeo T, Bianco GV, de CD, Aközbek N, Petruzzelli V, Bruno G, De VM, Scalora M, D’Orazio A (2014). Graphene-based absorber exploiting guided mode resonances in one-dimensional gratings. Opt Express.

[16] Zheng G, Zhang H, Xu L, Liu Y (2016). Enhanced absorption of graphene monolayer with a single-layer resonant grating at the Brewster angle in the visible range. Opt Lett.

[17] Guo CC, Zhu ZH, Yuan XD, Ye WM, Liu K, Zhang JF, Xu W, Qin AQ (2016). Experimental demonstration of total absorption over 99% in the near infrared for monolayer-graphene-based subwavelength structures. Adv Opt Mater.

[18] Long Y, Shen L, Xu H, Deng H, Li Y (2016). Achieving ultranarrow graphene perfect absorbers by exciting guided-mode resonance of one-dimensional photonic crystals. Sci Rep.

[19] Hu JH, Huang YQ, Duan XF, Wang Q, Zhang X, Wang J, Ren XM (2014). Enhanced absorption of graphene strips with a multilayer subwavelength grating structure. Appl Phys Lett.

[20] Piper JR, Fan S (2014). Total absorption in a graphene monolayer in the optical regime by critical coupling with a photonic crystal guided resonance. ACS Photon.

[21] Sang T, Wang R, Li J, Zhou J, Wang Y (2018). Approaching total absorption of graphene strips using a c-Si subwavelength periodic membrane. Opt Commun.

[22] Wang W, Klots A, Yang Y, Li W, Kravchenko II, Briggs DP, Bolotin KI, Valentine J (2015). Enhanced absorption in two-dimensional materials via Fano-resonant photonic crystals. Appl Phys Lett.

[23] Zheng G, Zou X, Chen Y, Xu L, Liu Y (2017). Tunable spectrum selective enhanced absorption of monolayer graphene in Fano resonant waveguide grating with four-part period. Plasmonics.

[24] Lu H, Cumming BP, Gu M (2015). Highly efficient plasmonic enhancement of graphene absorption at telecommunication wavelengths. Opt Lett.

[25] Lu H, Gan X, Jia B, Mao D, Zhao J (2016). Tunable high-efficiency light absorption of monolayer graphene via Tamm plasmon polaritons. Opt Lett.

[26] Cai Y, Zhu J, Liu QH (2015). Tunable enhanced optical absorption of graphene using plasmonic perfect absorbers. Appl Phys Lett.

[27] Zou X, Zheng G, Cong J, Xu L, Chen Y, Lai M (2018). Polarization-insensitive and wide-incident-angle optical absorber with periodically patterned graphene-dielectric arrays. Opt Lett.

[28] Liang J, Song X, Li J, Lan K, Li P (2017). A visible-near infrared wavelength-tunable metamaterial absorber based on the structure of Au triangle arrays embedded in VO_2_ thin film. J Alloys Comp.

[29] Zhou J, Yan S, Li C, Zhu J, Liu QH (2018). Perfect ultraviolet absorption in graphene using the magnetic resonance of an all dielectric nanostructure. Opt Express.

[30] Ustunsoy MP, Sabah C (2016). Dual-band high-frequency metamaterial absorber based on patch resonator for solar cell applications and its enhancement with graphene layers. J Alloys Comp.

[31] Liu B, Tang C, Chen J, Wang Q, Pei M, Tang H (2017). Dual-band light absorption enhancement of monolayer graphene from surface plasmon polaritons and magnetic dipole resonances in metamaterials. Opt Express.

[32] Wang R, Sang T, Wang L, Gao J, Wang Y, Wang J (2018). Enhanced absorption of a monolayer graphene using encapsulated cascaded gratings. Optik.

[33] Liu B, Tang C, Chen J, Xie N, Tang H, Zhu X, Gs P (2018). Multiband and broadband absorption enhancement of monolayer graphene at optical frequencies from multiple magnetic dipole resonances in metamaterials. Nanoscale Res Lett.

[34] Wang N, Bu L, Chen Y, Zheng G, Zou X, Xu L, Wang J (2017). Multiband enhanced absorption of monolayer graphene with attenuated total reflectance configuration and sensing application. Appl Phys Exp.

[35] Zheng G, Cong J, Chen Y, Xu L, Xiao S (2017). Angularly dense comb-like enhanced absorption of graphene monolayer with attenuated total reflection configuration. Opt Lett.

[36] Xiao G, Zhu Q, Shen Y, Li K, Liu M, Zhuang Q, Jin C (2015). A tunable submicro-optofluidic polymer filter based on guided-mode resonance. Nanoscale.

[37] Palik ED (1998) Handbook of optical constants of solids. Academic Press, pp 350–357

[38] Hanson GW (2008). Dyadic Green’s functions and guided surface waves for a surface conductivity model of graphene. J Appl Phys.

[39] Hanson GW (2008). Quasi-transverse electromagnetic modes supported by a graphene parallel-plate waveguide. J Appl Phys.

[40] Zhou L, Zhou Y, Zhu YF, Dong XX, Gao BL, Wang YZ, Shen S (2016). Broadband bidirectional visible light absorber with wide angular tolerance. J Mater Chem C.

[41] Wang H, Wang L (2013). Perfect selective metamaterial solar absorbers. Opt Express.

[42] Ye F, Burns MJ, Naughton MJ (2015). Symmetry-broken metamaterial absorbers as reflectionless directional couplers for surface plasmon polaritons in the visible range. Adv Opt Mater.

[43] Tian X, Li ZY (2016). Visible-near infrared ultra-broadband polarization-independent metamaterial perfect absorber involving phase-change materials. Photon Res.

[44] Song H, Zhang J, Fei G, Wang J, Jiang K, Wang P, Lu Y, Iorsh I, Xu W, Jia J, Zhang L, Kivshar YS, Zhang L (2016). Near-field coupling and resonant cavity modes in plasmonic nanorod metamaterials. Nanotechnology.

[45] Liu X, Gao J, Yang H, Wang X, Tian S, Guo C (2017). Hybrid plasmonic modes in multilayer trench grating structures. Adv Opt Mater.

[46] Zhao B, Zhang ZM (2014). Study of magnetic polaritons in deep gratings for thermal emission control. J Quant Spectrosc Radiat Transf.

[47] Wu YKR, Hollowell AE, Zhang C, Guo LJ (2013). Angle-insensitive structural colours based on metallic nanocavities and coloured pixels beyond the diffraction limit. Sci Rep.

[48] Shao H, Wang J, Liu D, Hu ZD, Xia X, Sang T (2017). Plasmonic planar lens based on slanted nanoslit array. Plasmonics.

[49] Kumar M, Tervo J, Kaplas T, Svirko Y (2016). Graphene-enhanced waveguide-resonance gratings. J Nanophotonics.

