# Supplementary material for: Angle-Insensitive Broadband Absorption Enhancement of Graphene Using a Multi-Grooved Metasurface
Source: Nanoscale Res Lett. 2019 Mar 20;14:105. doi: 10.1186/s11671-019-2937-7 (PMC6426901; doi:10.1186/s11671-019-2937-7)
Supplement: Supplementary file 1 — Figure S1. Absorption spectra of the total structure, graphene, and silver for the multi-grooved metasurface with graphene under the TE wave illumination. Figure S2 Absorption spectra for the graphene-based metasurface with different number of groove. (DOCX 380 kb) [file 11671_2019_2937_MOESM1_ESM.docx]

**Supplementary for “Angle-insensitive broadband absorption enhancement of graphene using a multi-grooved metasurface”**

Tian Sang^1*^, Jian Gao^1^, Xin Yin^1^, Honglong Qi^1^, La Wang^1^ and Hongfei Jiao^2*^

^1^Department of Photoelectric Information Science and Engineering, School of Science, Jiangnan University, Wuxi 214122, China

*^2^Key Laboratory of Advanced Micro-Structured Materials MOE, Institute of Precision Optical Engineering, School of Physics Science and Engineering, Tongji University, Shanghai 200092, China*

^*^Correspondence: sangt@jiangnan.edu.cn; jiaohf@tongji.edu.cn

Figure S1 shows the absorption spectra of the multi-grooved metasurface with graphene under the TE wave illumination (electric-field vector lies along the *y*-axis). As can be seen in Fig. S1, there is no obvious absorption enhancement in the whole visible region. This is because that the light absorption of the multi-grooved metasurface is related to the surface plasmon effect of the nanostructured silver film. However, surface plasmon mode can only be excited by the TM polarization, and it cannot be excited by the TE polarization [1,2]. Although the light energy is mainly dissipated in graphene rather than in silver, there is no absorption peak and the overall absorption is low due to the absence of surface plasmon.





**Fig. S1** Absorption spectra of the total structure, graphene and silver for the multi-grooved metasurface with graphene under the TE wave illumination. The parameters are: *Λ*=300 nm, *t*=5 nm, *w*=30 nm, *D*=100 nm, *d_1_*=20 nm, *d_2_*=35 nm, *d_3_*=50 nm, *d_4_*=80 nm, *d_5_*=90 nm, N=10, and *θ_c_*=0°.

Figure S2 shows the absorption spectra for the graphene-based metasurface with different number of groove under the TM wave illumination, and the width of each groove is fixed at 30 nm. As can be seen in Fig. S2(a), for the structure with three grooves, the absorption spectra of graphene is fluctuated and the absorption band is not flat in the whole visible region. This is because that the absorption peak of each groove are separated by a large distance in wavelength, and they cannot be well overlapped to form a broad and flat absorption band. However, see Fig. S2(b), as the number of the groove is increased to seven, the overall light absorption of graphene is reduced although its absorption band becomes flat. This may be resulted from the electromagnetic coupling between the neighboring grooves which are very close to each other. Thus it does not mean that the more the number of the groove is, the better the absorption performance of graphene will be.


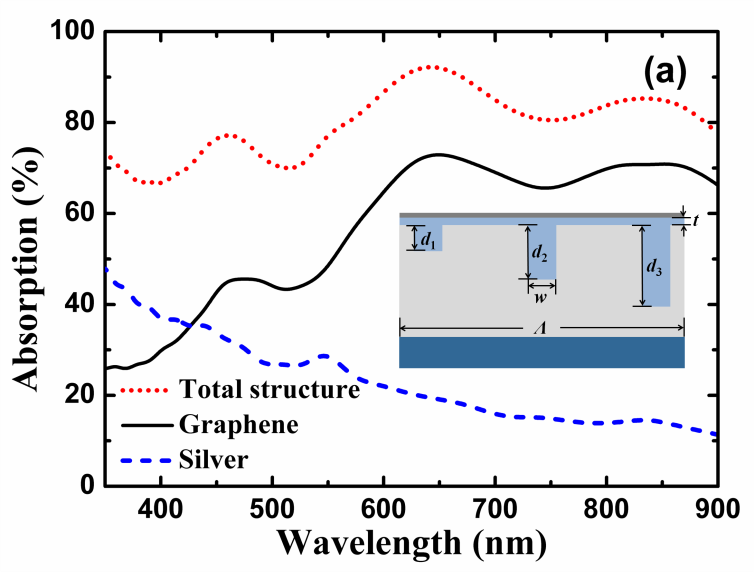


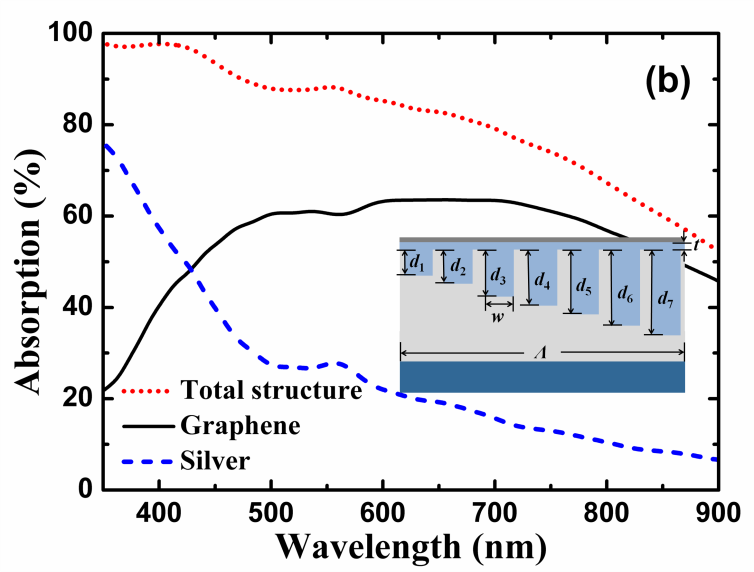


**Fig. S2** Absorption spectra for the graphene-based metasurface with different number of groove. (a) With three grooves, *d_1_*=20 nm, *d_2_*=55 nm, and *d_3_*=90 nm. (b) With seven grooves, *d_1_*=20 nm, *d_2_*=32 nm, *d_3_*=43 nm, *d_4_*=55 nm, *d_5_*=67 nm, *d_6_*=78 nm, and *d_7_*=90 nm. Other parameters are: *Λ*=300 nm, *t*=5 nm, *w*=30 nm, *D*=100 nm, N=10, and *θ_c_*=0°.

**References**

1. Barnes WL, Dereux A, Ebbesen TW (2003) Surface plasmon subwavelength optics. Science 424:824–830
2. Berini P (2009) Long-range surface plasmon polaritons. Adv Opt Photonics 1:484–588
